# Science and art in the 21st century: on a way toward the unification

**DOI:** 10.3325/cmj.2014.55.542

**Published:** 2014-10

**Authors:** Dinko Mitrečić, Franco Rustichelli

**Affiliations:** 1 Laboratory for Stem Cells, Croatian Institute for Brain Research, University of Zagreb School of Medicine, Zagreb, Croatia; 2 Department of Odontostomatologic and Specialized Clinical Sciences, Marche Polytechnic University, Ancona, Italy

When Paul Dirac, in 1928, published his famous equation in which he explained the link between the theory of relativity and the behavior of waves, thus predicting the presence of matter and antimatter (1), Albert Einstein said that Dirac, to invent this theory had to balance between the genius of the scientist and the madness of the artist. On the other hand, and more pessimistically, D.H. Lawrence said that by revealing this formula we had finally demystified the Universe. “We killed the sun, making it a ball of gas…” he cried (2).

Indeed, in the 21st century, like never before, a confusing dichotomy of Janus is confronted. While living in a time when we can enjoy the fruit of many past centuries’ different art styles, from the mysterious beauty of Gregorian chorales to the poetic glory of Picasso, it is potentially terrifying that our artistic sensuality could be accumulated to the point of absurdity. Thus Ardengo Soffici, the Italian futurist, said that the art’s final masterpiece is revealed in its own destruction (3).

In parallel, decoding the human genome apparently opened the book (Pandora box?) of absolute knowledge of ourselves. Given the still-not-insignificant time and money to read all three billion letters of a child’s complete genome, we could, for example, predict probability if the child will be talented enough to play the piano (4), what disease will affect him during his lifetime (5), if he would be prone to believe in God (6) and finally, theoretically, we may foresee a potential cause of his death. Concurrently, swift developments in stem cell research and the field of regenerative medicine have led to new possibilities in the treatment of currently incurable diseases, especially those affecting the nervous system (7). Moreover, new approaches offered the most intriguing possibility: if we ensure a constant source of rejuvenating stem cells, we will, theoretically, live forever (8). A beauty of this idea is hidden in its unmatched artistic simplicity: the question of life and death is shaken by a new era of medicine that has begun to build an immortal man. At the same time, a significantly improved understanding of the “artistic” and “human specific” regions of our brain have begun to motivate modern art performers to include science and scientific methods in the process of art production. Detection of electrical activity during muscle contraction can be transformed into colors or sounds, thus producing a multisensory experience during dance. So, can we exist as separate entities, scientists and artists, anymore?

With this and many other questions in mind, European Cooperation in Science and Technology (COST) fully supported the organization of a conference entitled Bridging the Gap Between Science and Art, which took place on May 12-14 in picturesque Monteconero near Ancona. Inspired by the above mentioned idea, in his essay about immortality, Franco Rustichelli, scientist and artist, raised provoking questions: Why the nature has not designed all women to look like Scarlett Johansson and intelligent like Marie Curie, and all men handsome like George Clooney and intelligent like Albert Einstein? The answer is akin to why we reasonably expect a broken leg to heal, but not a malignant tumor. Nature would like to heal everything, but it is not able. Nature is not God, it is still imperfect. Would nature, having succeeded over billions of years of evolution to produce a marvelous being, a beautiful girl with golden hair playing a Chopin nocturne, feel satisfaction witnessing her slow yet progressive physical decay, the corrosion of her beauty, and her eventual death? Is it plausible to consider that nature could have programmed all that with certain refined sadism? This scenario is even less justifiable if we replace the concept of nature with that of a divine being. From both a humanistic and philosophical point of view, physical decay is not something natural, in the sense of something desired and designed by nature, but rather it is something deeply unnatural, in the sense of a mechanism – and deeply unsatisfactory for nature itself. And that is where human intelligence comes on the stage. Analogous to the instincts, such as recognition of food and the drive to reproduce, which evolved to guarantee immortality of species, human intelligence naturally aims to ensure the immortality of individuals. Human intelligence needs to confront, by all means, including both science and art, the awareness of death, in order to be able to develop mechanisms to defeat it.

Following Franco Rustichelli`s introductory presentation, the most intriguing dialogue between gathered scientists and artists, some of whom would not dare to take a side, occurred. Claud Hesse, visual artist, presented her research about artistic elements of DNA, where the borders between cold facts and imaginative force of art have been revealed as blurred and unnecessary. Intriguing questions and some even more intriguing answers came from Silke Britzen, an astronomer and expert in black holes (“both dark and holes, but still so attractive”). Faced with the incongruous concept of singularity, a point where immeasurably high mass is concentrated in immeasurably small volume, where no consistent physical rules exist, only a paradigmatic shift from the purely scientific toward a blossoming artistic idea can save us from vanishing into the madness of unknown. Many of these ideas came out of a surprising explanation of what Michelangelo actually painted on the ceiling of the Sistine chapel: if inspected carefully, perhaps God is indeed sitting not in the a mass resembling the brain, but in probably the most intriguing organ on the earth: the placenta, the organ painted centuries earlier by Egyptian painters among symbols of pharaoh glory. Angels’ heads represent cotyledons of a placenta and the famous touch between God’s and Adam’s fingers the connection of the umbilical cord that delivers life from the mother to the unborn child (9). But when we examine it carefully, we actually do not see the touch of life, but rather a gently liberating gesture. Like a new life that becomes independent when the umbilical cord is cut, God does not touch Adam, but softly sends him away. In this painting we discover a pure neoplatonic idea: the creative source of everything is beyond being, in both power and dignity. The uppermost truth cannot be reached, but can be presented by a work of art. And that’s where science needs art and art needs science.

To strengthen the connection between art and science, Cristina Stefanon, pianist, presented her idea that the mystery of music can be revealed in beauty of mathematics. Although mathematics cannot reveal all the secrets of the inversions of Bach’s fugues or the intuitive logic of the dodecaphonic music system, it has opened new horizons to a better understanding of both art and science: it is in between moments of silence, in between mathematical equations, where the beauty sleeps. To close the discussion of the secrets of beauty and love, Dinko Mitrečić, neuroscientist, revealed a medical fact with intriguing artistic significance: neurons, cells that comprise neuronal networks, the configuration and extent of which constitute the critical difference between human and animals, depend on something: to survive, they must forge synaptic contacts with each other. No neuron can survive as an island, no neuron can survive without reaching for another neuron, or many others (10). The strength of those connections, measurable by scientific parameters, reveals which biologic elements allow humans to be humans, to build consciousness and to be able to express love for others. Like the connection between an unborn child and his parents, partly hidden in an inscrutable, indescribable mist, but miraculously revealed in growing anticipation and visionary dreams, our spiritual, mental, and physical potential can be fully reached by letting the energy of art and science dance together and bring us closer to understanding – where we are and where we are heading.

From the microscopic, cellular level to the level of societal networks, from science to art and back – whatever side we take as a starting point, there is always the immortal force that motivates humans to better understand and better express the beauty hidden in everything around us. Indeed, only beautiful science can be important and only art which intrigues the mind can be beautiful. When taken together, the gap between art and science is bridged, again and again, by profound connections which imbue our lives with meaning.
